# Characterization of Stimulus-Secretion Coupling in the Human Pancreatic EndoC-βH1 Beta Cell Line

**DOI:** 10.1371/journal.pone.0120879

**Published:** 2015-03-24

**Authors:** Lotta E. Andersson, Bérengère Valtat, Annika Bagge, Vladimir V. Sharoyko, David G. Nicholls, Philippe Ravassard, Raphael Scharfmann, Peter Spégel, Hindrik Mulder

**Affiliations:** 1 Department of Clinical Sciences, Unit of Molecular Metabolism, Lund University Diabetes Centre, CRC, Malmö, Sweden; 2 Buck Institute for Research on Aging, Novato, California, United States of America; 3 Université Pierre et Marie Curie-Paris 6, Biotechnology and Biotherapy Team, Centre de Recherche de I’Institut du Cerveau et de la Moelle épiniére (CRICM), UMRS 975, Paris, France; 4 INSERM U1016, Cochin Institute, Université Paris Descartes, Sorbonne Paris Cité, Faculté de Médecine, Faculty Cochin, Paris, France; Broad Institute of Harvard and MIT, UNITED STATES

## Abstract

**Aims/Hypothesis:**

Studies on beta cell metabolism are often conducted in rodent beta cell lines due to the lack of stable human beta cell lines. Recently, a human cell line, EndoC-βH1, was generated. Here we investigate stimulus-secretion coupling in this cell line, and compare it with that in the rat beta cell line, INS-1 832/13, and human islets.

**Methods:**

Cells were exposed to glucose and pyruvate. Insulin secretion and content (radioimmunoassay), gene expression (Gene Chip array), metabolite levels (GC/MS), respiration (Seahorse XF24 Extracellular Flux Analyzer), glucose utilization (radiometric), lactate release (enzymatic colorimetric), ATP levels (enzymatic bioluminescence) and plasma membrane potential and cytoplasmic Ca^2+^ responses (microfluorometry) were measured. Metabolite levels, respiration and insulin secretion were examined in human islets.

**Results:**

Glucose increased insulin release, glucose utilization, raised ATP production and respiratory rates in both lines, and pyruvate increased insulin secretion and respiration. EndoC-βH1 cells exhibited higher insulin secretion, while plasma membrane depolarization was attenuated, and neither glucose nor pyruvate induced oscillations in intracellular calcium concentration or plasma membrane potential. Metabolite profiling revealed that glycolytic and TCA-cycle intermediate levels increased in response to glucose in both cell lines, but responses were weaker in EndoC-βH1 cells, similar to those observed in human islets. Respiration in EndoC-βH1 cells was more similar to that in human islets than in INS-1 832/13 cells.

**Conclusions/Interpretation:**

Functions associated with early stimulus-secretion coupling, with the exception of plasma membrane potential and Ca^2+^ oscillations, were similar in the two cell lines; insulin secretion, respiration and metabolite responses were similar in EndoC-βH1 cells and human islets. While both cell lines are suitable *in vitro* models, with the caveat of replicating key findings in isolated islets, EndoC-βH1 cells have the advantage of carrying the human genome, allowing studies of human genetic variants, epigenetics and regulatory RNA molecules.

## Introduction

Defective insulin secretion by pancreatic beta cells underlies type 2 diabetes mellitus (T2D), a disease that increases globally and soon is estimated to affect >500 million people [1]. Despite decades of research, neither the regulation of insulin secretion nor the mechanism underlying the disease is completely understood.

Stimulus-secretion coupling in the beta cell links a rise in postprandial blood glucose levels to insulin release. Glucose is transported into the beta cell and metabolized to yield pyruvate, which in turn is further metabolized to raise ATP-levels [2]. This increase in the ATP/ADP-ratio closes ATP-dependent K^+^-channels (K^+^_ATP_-channels) in the plasma membrane [2]. Closure of K^+^-channels depolarizes the cell membrane, causing an opening of voltage-gated Ca^2+^-channels and release of insulin [3]. This pathway, known as the triggering pathway, is complemented by an amplifying pathway [4]. Numerous studies have been devoted to elucidate the nature of the latter enigmatic pathway [5].

Stimulus-secretion coupling has primarily been studied in insulinoma cell lines and rodent isolated islets. These studies imply differences between species as well as between clonal and primary cells. In recent times, human islets have been made available to research, but their number is limited. In addition to beta cells, islets also contain significant numbers of α-, δ-, PP, ε-cells and blood vessel endothelial cells [6,7], limiting the use of islets as a specific beta cell model. Moreover, rodent and human beta cells and islets show differences in the expression of key enzymes in glucose metabolism, in the insulin gene (two genes in rodents while one gene in humans) [8], glucose transporters [9], and islet structure [10]. Attempts have been made to develop human beta cell lines; however, these lines show low levels of insulin production, slow growth rate or limited phenotypic and functional stability [11,12]. Recently, a stable human beta cell line, EndoC-βH1, was derived using targeted oncogenesis in human fetal pancreatic tissue [13]. EndoC-βH1 cells produce and secrete insulin in response to glucose, are stable in culture and express beta cell-specific markers, such as PDX1 and MAFA. Transplantation of EndoC-βH1 cells reinstated normoglycemia in STZ-induced diabetic mice [13].

In the present study, we attempted to provide a comprehensive characterization of stimulus-secretion coupling in the EndoC-βH1 beta cell line by comparing glucose metabolism in this cell line and in the clonal rat cell line, INS-1 832/13 [14,15]. Key experiments were repeated in isolated human islets.

## Methods

### *In vitro* models and human islets

EndoC-βH1 cells (EndoCells, Paris, France) [13] were grown on Matrigel-fibronectin coated (100 μg/mL and 2 μg/mL, respectively, Sigma-Aldrich, Steinheim, Germany) culture vessels in DMEM containing 5.6 mM glucose, 2% BSA fraction V (Roche Diagnostics, Mannheim, Germany), 10 mM nicotinamide (Merck Millipore, Darmstadt, Germany), 50 μM 2-mercaptoethanol, 5.5 μg/mL transferrin, 6.7 ng/mL sodium selenite (Sigma-Aldrich), 100 U/mL penicillin, and 100 μg/mL streptomycin (PAA Laboratories, Pasching, Austria). INS-1 832/13 cells were cultured as previously described [14]. Both cell lines were cultured at 37°C in air with 5% CO_2_. Cell viability was assessed by trypan blue exclusion. Unless otherwise stated, EndoC-βH1 cells were seeded at 2.3x10^5^ cells/cm^2^ and INS-1 832/13 cells at 1.5x10^5^ cells/cm^2^ in 24-well plates (Matrigel-fibronectin coated or uncoated) and cultured for 72 hours followed by an overnight pre-incubation in 2.8 mM glucose media (starvation media) before assays were performed. Human islets, isolated and treated as previously described [16], from non-diabetic donors (aged 61.1±3.3 years, BMI of 26.9±0.8, HbA_1c_ of 5.9±0.1) were supplied from the Human Tissue Laboratory at Lund University Diabetes Centre, which receives islets on a regular basis from the Nordic Center For Clinical Islet Transplantation (Uppsala, Sweden; Professor Olle Korsgren). Experimental procedures were approved by the Lund University Ethical Board. The procedure adhered to the Declaration of Helsinki (2000) and the World Medical Association.

### Insulin secretion, insulin content and lactate secretion

EndoC-βH1 cells were incubated in HEPES-buffered Krebs-Ringer Buffer (KRB—115 mM NaCl, 24 mM NaHCO_3_, 5 mM KCl, 1 mM MgCl_2_, 1 mM CaCl_2_, 10 mM HEPES, 0.2% BSA, pH 7.4) and INS-1 832/13 cells in HEPES-balanced salt solution (HBSS) [17] containing 0.5 mM glucose for 1 hour. Finally, cells were incubated in KRB or HBSS containing 1 or 20 mM glucose supplemented with either 5 or 35 mM KCl, or 10 mM pyruvate for 1 hour. Insulin secretion from 300 human islets was determined as previously described in detail [18]. Acid-Ethanol extraction was used for insulin content [19]. Insulin secretion and content were measured by the Coat-a-Count radioimmunoassay (RIA) (Siemens Medical Solutions Diagnostics, Los Angeles, CA) according to manufacturer’s instructions. Lactate released from cells was measured using a colorimetric lactate assay kit (BioVision, San Francisco, CA).

### Gene Expression

mRNA levels of genes important to cellular metabolism in INS-1 832/13 cells were determined by the Gene Chip Rat Gene 1.ST Array (Affymetrix, Santa Clara, CA) [17]. Expression levels in EndoC-βH1 cells were determined by the Gene Chip Human Genome U133 Plus 2.0 Array (Affymetrix).

### RNA isolation and Quantitative real-time PCR

Total RNA was extracted from EndoC-βH1 cells, INS-1 832/13 cells and human islets using TRI Reagent (Sigma Aldrich) according to manufacturer’s protocol. RNA concentrations were determined using a NanoDrop Spectrophotometer (Thermo Scientific). Equal quantities of total RNA were reverse transcribed using RevertAid First-Strand cDNA synthesis kit (Fermentas, Vilnius, Lithuania) in reactions containing 500 ng of total RNA. Quantitative real-time PCR (Q-PCR) was performed using the TaqMan gene expression assay (CACNA1A/Ca_v_1.2: Hs00930488, Rn00709287; CACNA1C/Ca_v_1.3: Hs00167753, Rn01453378; CACNA1D/Ca_v_2.1: Hs01579431, Rn00563825; CACNA1H/Ca_v_3.2: Hs00234934, Rn01460348; Assay on demand, Applied Biosystems, Life Technologies, Carlsbad, CA), using a 7900HT Fast Real-Time System (Applied Biosystems). The qPCR was carried out as previously described [20]. Gene expression was quantified by the comparative Ct method, in which the amount of target is expressed as 2^-ΔΔCt^ using hypoxanthine-guanine phosphoribosyl transferase (HPRT1) as reference gene.

### Metabolite profiling

Metabolism of cells and islets from the insulin secretion assay was quenched by adding 70 μL ice-cold Milli-Q water and 300 μL ice-cold extraction solvent, respectively [21]. Metabolites were extracted and derivatized as previously described [21,22]. Metabolite extracts were analyzed on an Agilent 6890N gas chromatograph (Agilent Technologies, Atlanta, GA) equipped with an Agilent 7683B auto-sampler (Agilent Technologies) and coupled to a LECO Pegasus III TOFMS electron impact time-of-flight mass spectrometer (LECO Corp., St. Joseph, MI) as previously described [23].

### Respiration

Oxygen consumption rates (OCR) were measured by the XF24 Extracellular Flux Analyzer (Seahorse Bioscience, North Billerica, MA) as previously described [17]. Cells or human islets were pre-incubated for 1 hour at 37°C in air after which respiration was measured in the presence of 1 mM glucose, 20 mM glucose or for cells lines also 10 mM pyruvate. Oligomycin, carbonyl cyanide-p-trifluoromethoxy-phenylhydrazone (FCCP) and rotenone were injected as described previously unless otherwise stated [17,24]. All calculations were done as previously described [25].

### Glucose utilization

Cells were incubated in KRB or HBSS containing D-[5-^3^H] glucose and glucose to a final concentration of 1 or 20 mM glucose. Glycolytic rate was estimated from the rate of [^3^H]OH production from D-[5-^3^H]glucose, as measured by liquid scintillation [17].

### ATP levels

Cells were lysed by 200 mM NaCl, 2 mM EDTA, 50 mM Tris and 1% Triton X-100 (pH 7.4) followed by flash-freezing on dry-ice/ethanol. ATP was assayed using a luciferase-based luminescent assay (BioTherma, Handen, Sweden) according to manufacturer’s instructions.

### Plasma membrane potential changes

Cells were seeded onto Matrigel-fibronectin coated 8-well chambered cover glasses (Lab-Tek, Naperville, IL) and incubated overnight in starvation medium, followed by incubation in 400 μL of buffer P (135 mM NaCl, 3.6 mM KCl, 1.5 mM CaCl_2_, 0.5 mM MgSO_4_, 0.5 mM Na_2_HPO_4_, 10 mM HEPES, 5 mM NaHCO_3_, pH 7.4) containing 2.8 mM glucose for 2 hours. A vial from a FLIPR membrane potential assay kit, explorer format component A, containing a proprietary plasma membrane potential (Δψ_p_) indicator (“PMPI”) (R-8042; Molecular Devices, Sunnydale, CA) was reconstituted in 10 mL water, and 4 μL added to the incubation immediately prior to imaging as described previously [26,27]. Excitation was performed at 514 nm and emission recorded with a 530 nm long-pass filter [26] on a Zeiss LSM510 inverted confocal fluorescence microscope.

### Cytoplasmic free Ca^2+^

After pre-incubation in starvation media, cells were incubated in 400 μL of buffer P. After 1.5 hours, 2 μM Fluo-4 AM (Invitrogen, Life Technologies, Carlsbad, CA), 0.25 mM sulfinpyrazone (a multi-specific inhibitor of organic anion transporters) and BSA (1 mg/mL) were added and the incubation was continued for a further 30 min. Finally, Fluo-4 AM was excited at 488 nm and emission recorded at 505–530 nm. Free cytoplasmic Ca^2+^ traces were displayed in arbitrary fluorescent units.

### Statistical analysis

Data are shown as means ± S.E.M. for the indicated number of experiments. Unless otherwise stated, paired Student’s *t*-test was used to compare differences between stimulation conditions and either a Student’s *t*-test or a Mann-Whitney U-test if variances were significantly different was used to compare differences between cell lines. Seahorse data were analyzed with Kruskal-Wallis tests and Dunn’s multiple comparisons test or Mann-Whitney U-tests as well as principal component analysis (PCA). Mean-centered and unit-variance scaled normalized metabolite data were analyzed in SIMCA-P^+^ 12.0 (Umetrics, Umeå, Sweden) by PCA and orthogonal projections to latent structures discriminant analysis (OPLS-DA).

## Results

### Proliferation, viability and insulin secretion

The INS-1 832/13 cells proliferated at a higher rate than EndoC-βH1 cells. The average doubling time of EndoC-βH1 cells was 174 hours compared to 44 hours for INS-1 832/13 cells. The viability in both cell lines was similar; approximately 95% (S1 Fig.).

**Fig 1:**
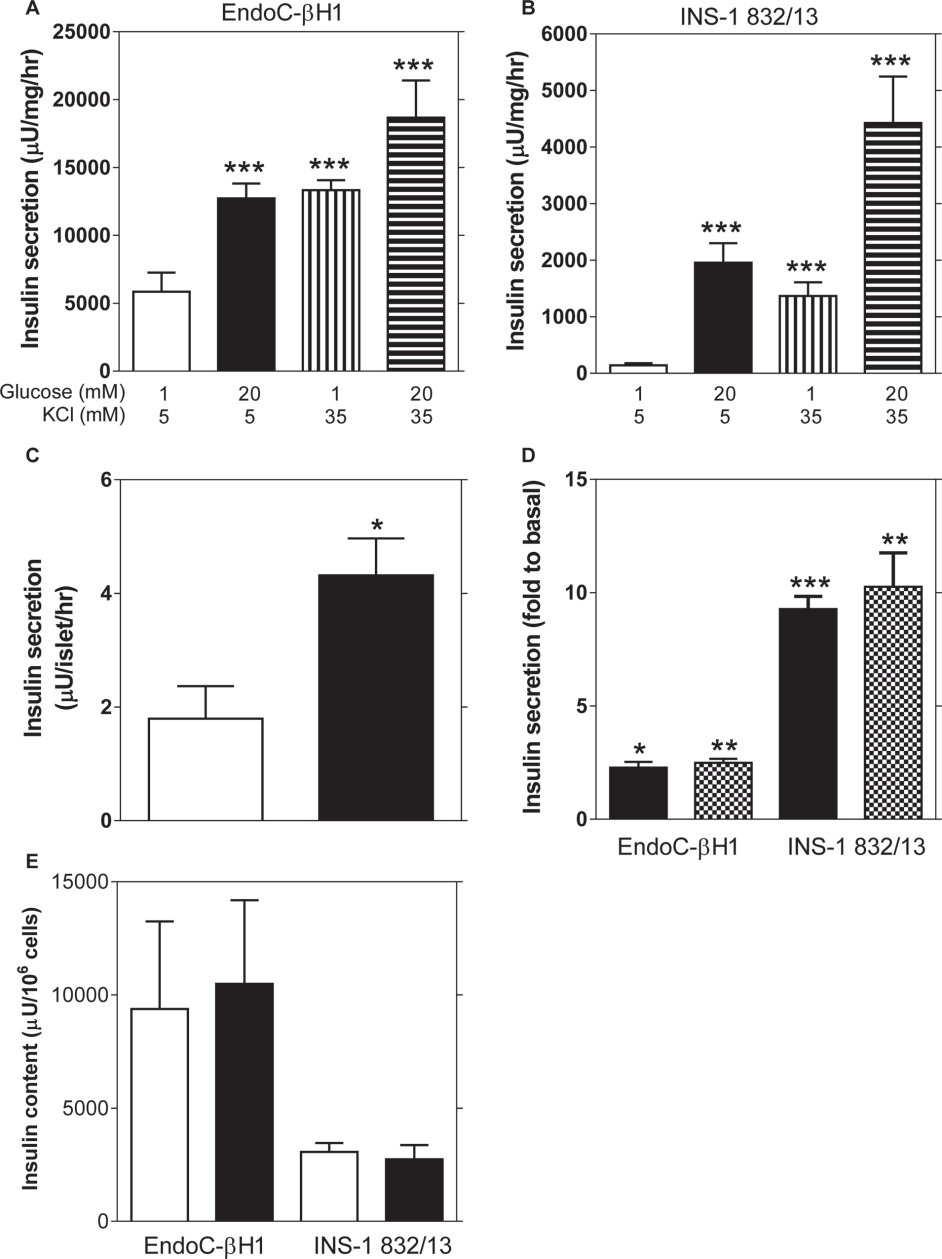
Glucose-stimulated insulin secretion in EndoC-βH1, INS-1 832/13 cell lines and isolated human islets. Basal (1 mM glucose) and glucose-stimulated (20 mM glucose) insulin secretion in EndoC-βH1 (A) and INS-1 832/13 cells (B) in the presence of 5 mM or 35 mM KCl. (C) Basal (2.8 mM glucose, white bar) and glucose-stimulated (16.7 mM glucose, black bar) insulin secretion in isolated human islets (n = 14 donors). (D) Insulin secretion after stimulation with 20 mM glucose (black bar) or 10 mM pyruvate (checkered bar) in both cell lines. (E) Total insulin content was evaluated as the sum of the intracellular and secreted insulin after basal (1 mM glucose, white bar) or glucose stimulated (20 mM glucose, black bar) insulin secretion for both cell lines. Data are expressed as mean ±S.E.M (n = 3, EndoC-βH1 and n = 4, INS-1 832/13). Differences within cell line were assessed by the paired Student’s t-test. *p<0.05, **p<0.01, ***p<0.001.

Stimulation of EndoC-βH1 and INS-1 832/13 cells with 20 mM glucose provoked a 2.4-fold and a 9.2-fold increase in insulin release, respectively. Basal and stimulated insulin secretion were 19-fold and 5-fold higher in EndoC-βH1 compared to INS-1 832/13 cells. KCl raised insulin secretion 2.8-fold and 4.2 fold at low and high glucose, respectively, in EndoC-βH1 compared with 7.2-fold and 23.4-fold in INS-1 832/13-cells (Fig. 1A,B). Glucose (16.7 mM) provoked a 2.4-fold increase in insulin secretion from human islets (Fig. 1C), similar to the fold increase of EndoC-βH1 cells. Pyruvate stimulation induced a 2.5-fold and 10.3-fold increase in insulin secretion in EndoC-βH1 and INS-1 832/13 cells, respectively (Fig. 1D). Insulin content was more than 3-fold greater in EndoC-βH1 than in INS-1 832/13 cells (Fig. 1E). The fold-responses to glucose stimulation were higher in all models when calculations were based on individual experiments rather than averaged data, as presented in the article (S2 Fig.). This was due to variability in insulin output at basal and stimulated conditions between experiments; this was particularly evident in the human islets where the stimulated output between islets batches ranged from 1.1 to 9.8 μU/islet/hr.

### Gene Expression

Next, mRNA expression of key metabolic enzymes and calcium channels was determined. The analysis revealed that out of the 41 genes examined all were expressed in INS-1 832/13 cells except G6PC2, while in EndoC-βH1 cells nine were not expressed (SLC2A4, HK1/2/3, LDHC, SLC1A3, PCK1, G6PC, ALDOB), and two genes (LDHD, G6PC3) presented inconclusive results (S1 Table). qPCR analysis of four voltage dependent calcium channels (CACNA1A/Ca_v_1.2, CACNA1C/Ca_v_1.3, CACNA1D/Ca_v_2.1, CACNA1H/Ca_v_3.2) revealed differential expression between INS-1 832/13 cells and EndoC-βH1 cells for CACNA1C and between INS-1 832/13 cells and both EndoC-βH1 and human islets for CACNA1H (S3 Fig.).

### Metabolite profiling

**Fig 2:**
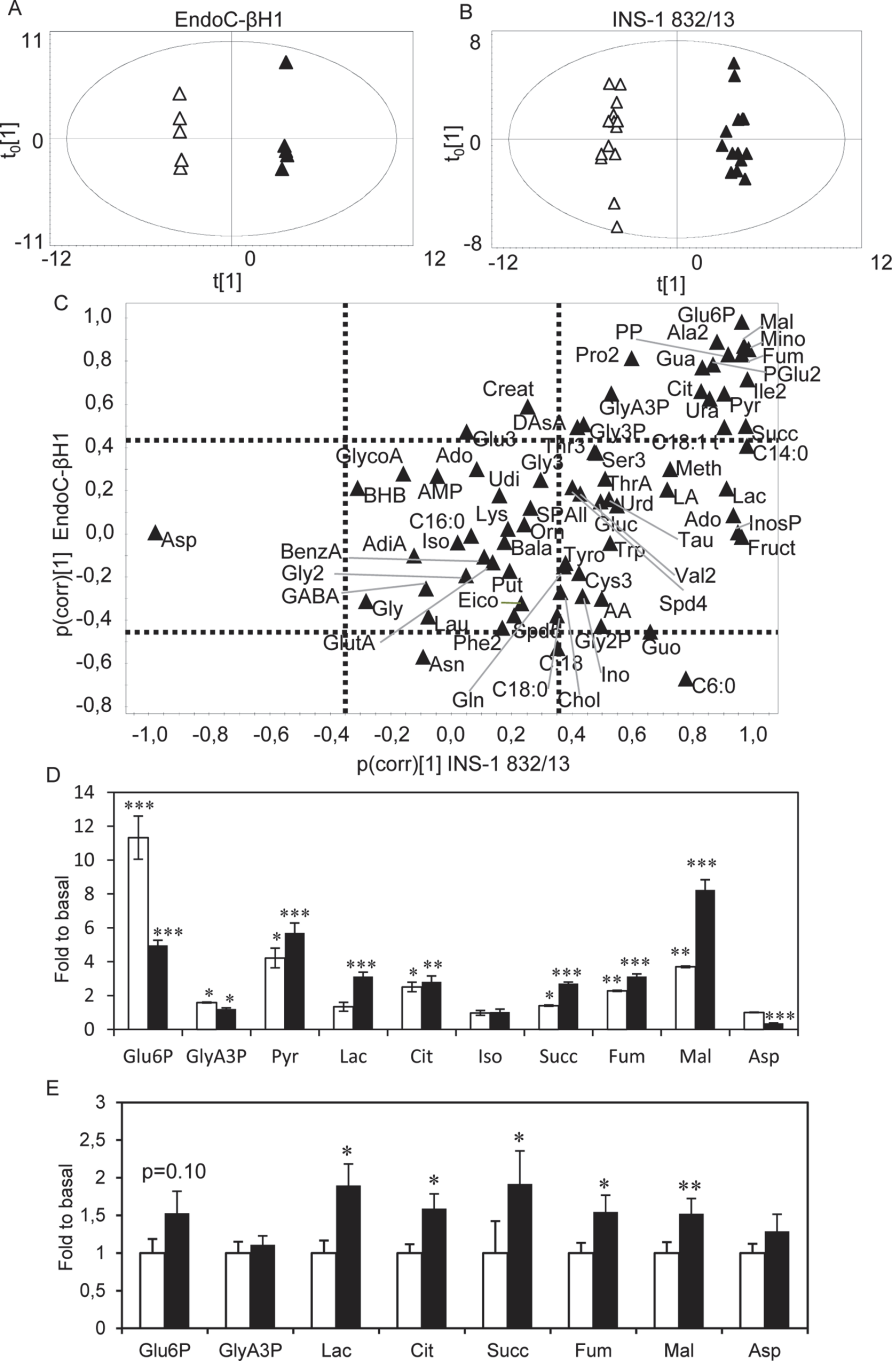
Metabolite levels after glucose stimulation in EndoC-βH1, INS-1 832/13 cells and isolated human islets. Score scatter plots of the metabolite profiles for (A) EndoC-βH1 and (B) INS-1 832/13 cells upon glucose stimulation with 1 mM (white triangles) or 20 mM (black triangles) glucose. (C) A SUS-like plot revealing alterations in metabolite levels after glucose stimulation underlying the clustering observed in the score-scatter plots in two dimensions. Dashed lines indicate significance levels; metabolites on the top and right sides are significantly increased while those on the bottom and left side are significantly decreased according to the cell type on the x and y-axis. Hence, metabolites in the upper right and lower left quadrants are up- and down-regulated, respectively, in both cell lines. Metabolites found in the middle right and left quadrants are up- and down-regulated, respectively, only in the INS-1 832/13 cells and those in the upper and lower centered quadrants are increased and decreased, respectively, after glucose stimulation in EndoC-βH1 cells. Metabolites in the center of the plot are unchanged. (D) Levels of glycolytic and TCA-cycle intermediate metabolites in 20 mM glucose relative to 1 mM glucose in EndoC-βH1 (white bars) and INS-1 832/13 (black bars) cells. (E) Relative levels of metabolites in 16.7 mM glucose relative to 2.8 mM glucose in isolated human islets. Data are expressed as mean ±S.E.M (n = 6 for cell lines, n = 14 for donors). Differences within cell line were assessed by the paired Student’s t-test. *p<0.05, **p<0.01, ***p<0.001.

Microarray and qPCR analysis of the cell lines revealed expression of virtually the same metabolic enzymes (S1 Table). To further assess metabolic regulation, metabolites were profiled at 1 mM and 20 mM glucose. In this analysis, 74 metabolite derivatives were identified, corresponding to 68 unique metabolites. Data were analyzed separately for the two cell lines, using orthogonal projections to latent structures—discriminant analysis (OPLS-DA) [28]. In these analyses, systematic variation in metabolite levels unrelated to the glucose stimuli as well as noise are removed; thereby isolating variation associated with the glucose stimuli. Consequently, the original 74 dimensions, defined by the number of detected metabolite derivatives, were reduced to one dimension (the predictive component). In addition, the contribution of all metabolites to the glucose elicited metabolic response is isolated. The score scatter plots, where the position of each point was determined by levels of all detected metabolite derivatives in a sample, revealed a perfect separation of samples from low and high glucose stimulated cells (Fig. 2A, 2B). This means that glucose stimulation provoked a profound and systematic shift in metabolism in both cell lines.

Loadings for the predictive components of the two models, scaled as correlations, were plotted in a shared-and-unique-structures (SUS)-like plot (Fig. 2C). Thereby, glucose- elicited changes in metabolite levels similar (shared) or different (unique) between the two cell-lines can be identified [29]. Metabolites displaying significantly altered levels were identified from the loading plots with jack-knifed confidence intervals. This plot revealed that the overall metabolic response to glucose stimulation was similar; regulation of the majority of metabolite levels was shared between the two cell lines. In both cell lines, glucose increased glycolytic and tricarboxylic acid (TCA)-cycle intermediates levels, such as glucose-6-phosphate (Glu6P; 11.3-fold for EndoC-βH1 vs. 5-fold for INS-1 832/13) and glyceric acid 3-phosphate (GlyA3P; 1.6-fold vs. 1.2-fold, respectively) (Fig. 2D). However, some metabolites were uniquely regulated in one of the cell lines; increased intracellular lactate (Lac; 3.1-fold) and reduced aspartate level (Asp; 2.8-fold) were observed only in INS-1 832/13 cells. Both cell lines expressed lactate dehydrogenase (LDH), and critical enzymes and carriers in the malate-aspartate- and glycerolphosphate-shuttles (S1 Table).

To investigate whether the differences in glycolytic and TCA-cycle metabolism observed between the rodent and human cell lines were cell line or species-specific, we measured the metabolic response to glucose stimulation in human islets (Fig. 2E). Overall, fewer metabolites were detectable with our GC/MS approach. Only two glycolytic intermediates (Glu6P and GlyA3P) were observed; increases were not significant. In contrast, more TCA-cycle intermediates were observed, and their levels generally increased significantly upon glucose stimulation. Also lactate levels were found to increase after glucose stimulation.

### Respiration

**Fig 3:**
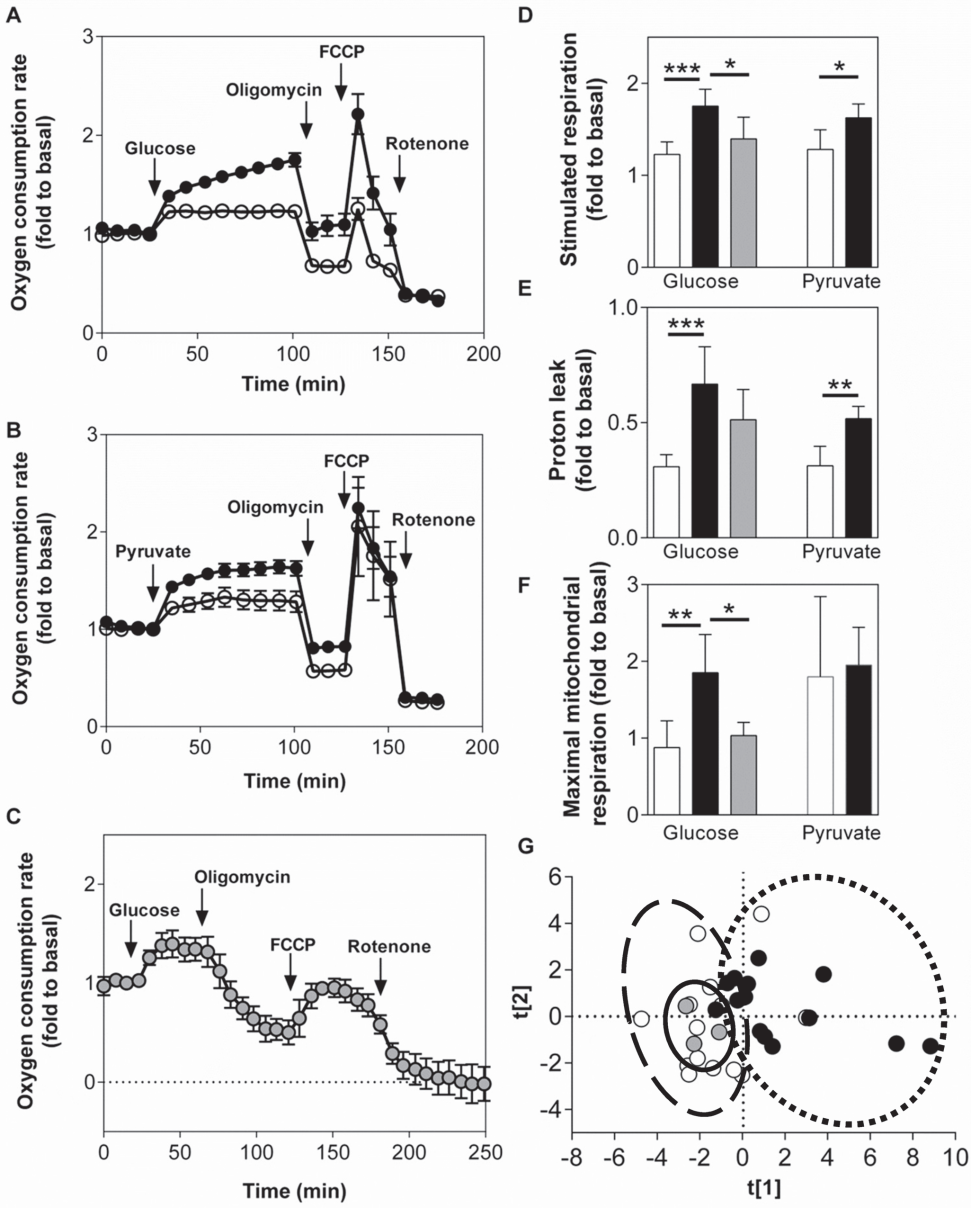
Respiration in EndoC-βH1, INS-1 832/13 cells and human islets. Oxygen consumption rates relative to basal (1 mM glucose) OCR upon glucose stimulation (20 mM; A, C) or pyruvate stimulation (10 mM; B) in EndoC-βH1 cells (A, B; white symbols), INS-1 832/13 cells (A, B; black symbols) and human islets (C; grey symbols). Glucose- and pyruvate-stimulated respiratory response (D), proton leak (oligomycin-insensitive glucose-stimulated respiration) (E) and maximal mitochondrial respiration (F) each expressed as fold relative to basal. (G) Principal component analysis of respiratory parameters (EndoC-βH1—dashed line, INS-1 832/13—dotted line, human islets—solid line) (PCA: R2X = 0.896; R2Y = 0.684; A = 3). All calculations were done after subtracting non-mitochondrial respiration. Data are represented as mean ±S.E.M (n = 8 for glucose, n = 4 for pyruvate and n = 3 for human islets). Statistical analysis was done as described in methods. *p<0.05, **p<0.01, ***p<0.001.

Since glucose- and pyruvate-stimulated insulin secretion was found to differ between the cell lines and human islets, we investigated whether this was associated with altered respiration. Respiration increased in both cell lines as well as in human islets in response to glucose (Fig. 3A, 3C). After correction for non-mitochondrial respiration, INS-1 832/13 cells displayed a significantly greater relative respiratory response to glucose (1.8-fold) compared to EndoC-βH1 cells (1.2-fold) (Fig. 3A, 3D), while the glucose response in human islets was similar to the response from EndoC-βH1 cells (Fig. 3C, 3D). INS-1 832/13 cells also showed a greater response to pyruvate (1.6-fold) compared to EndoC-βH1 cells (1.3-fold) (Fig. 3B). The relative proton leak was higher in INS-1 832/13 cells compared to EndoC-βH1 cells in the presence of elevated stimulatory glucose or pyruvate levels, while the proton leak in human islets did not differ from either of the cell lines (Fig. 3E). The coupling efficiency did not differ between the two cell lines or the human islets (data not shown). Glucose-stimulated EndoC-βH1 cells and human islets showed an attenuated maximal mitochondrial respiration rate (response to FCCP following oligomycin) compared to the INS-1 832/13 cells, whereas the relative response to FCCP of pyruvate-stimulated cells was comparable between the two cell lines (Fig. 3A, 3B, 3F). A PCA performed on the respiration data showed that EndoC-βH1 cells were more similar to human islets than to INS-1 832/13 cells (Fig. 3G).

### Glucose utilization, lactate and ATP production in EndoC-βH1 and INS-1 832/13-cells

**Fig 4:**
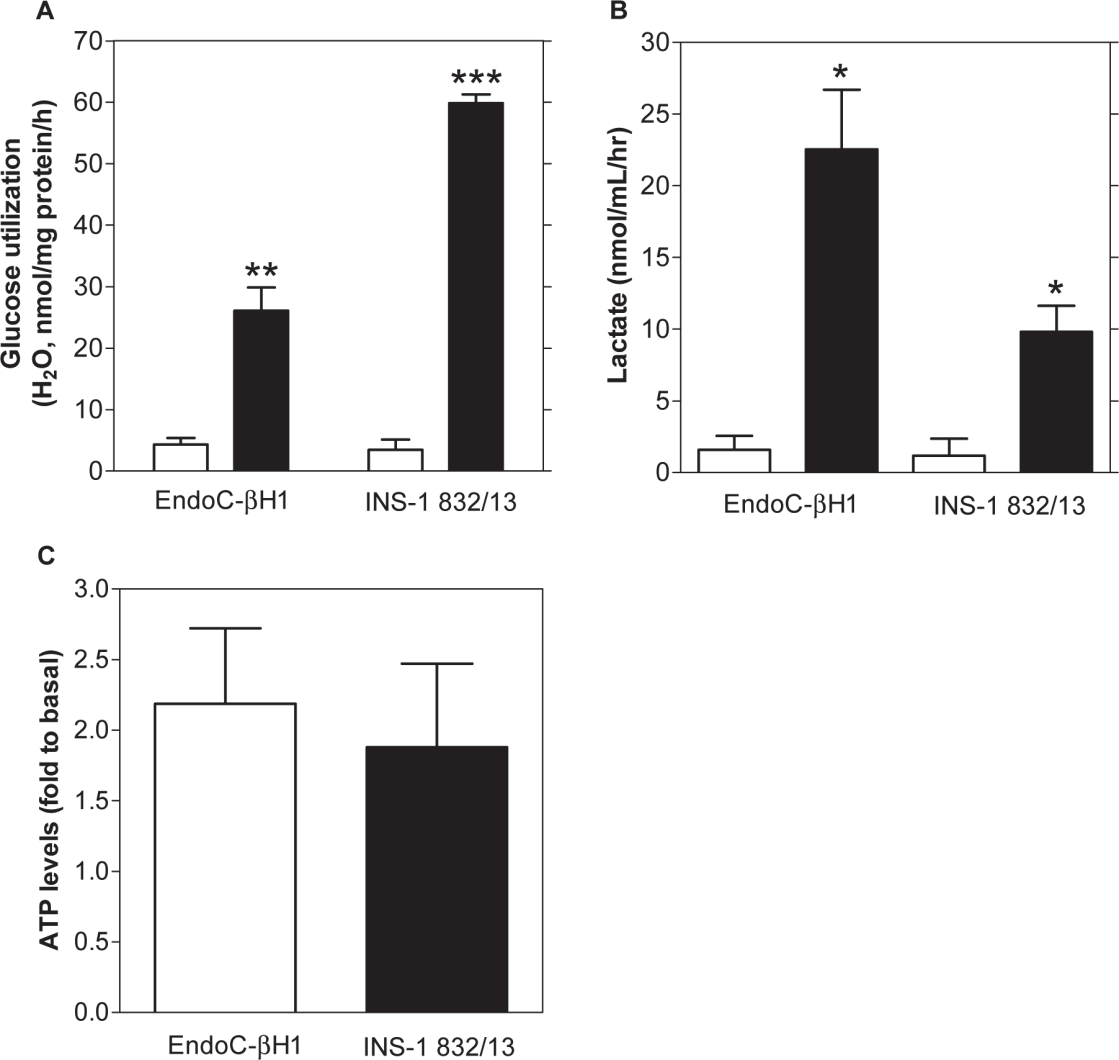
Glucose utilization, lactate and ATP levels in EndoC-βH1 and INS-1 832/13 cells. Glucose utilization (A) and extracellular lactate levels (B) in EndoC-βH1 cells in basal (1 mM glucose, white bars) and glucose-stimulated (20 mM glucose, black bars) conditions. Relative intracellular ATP levels (C) after glucose stimulation in EndoC-βH1 (white bars) and INS-1 832/13 (black bars) cells. Data are expressed as mean ±S.E.M (n = 3–6). Differences within cell line were assessed by a paired Student’s t-test. *p<0.05, **p<0.01, ***p<0.001.

Metabolite profiling revealed increases of aspartate and lactate levels in INS-1 832/13 cells in response to glucose. This suggests that cytosolic replenishment of NAD^+^ may differ between INS-1 832/13 and EndoC-βH1 cells. Since this process is critical to maintain a high glycolytic rate, we next investigated the flux of glucose through glycolysis. Basal glucose utilization was similar between the cell lines (Fig. 4A). Glucose stimulation provoked a 6-fold and 26-fold increase in glucose utilization in EndoC-βH1 and INS-1 832/13-cells, respectively.

Since both cell lines expressed LDH (S1 Table), we investigated lactate release from the cells. Basal lactate levels were similar, albeit near the limit of detection of the assay whereas glucose-stimulated lactate release increased 14.3-fold and 8.3-fold in EndoC-βH1 and INS-1 832/13-cells, respectively (Fig. 4B).

Next, we analyzed whether the differences observed in the rate of glucose metabolism and respiration may be translated into differences in ATP levels, the main trigger of glucose-stimulated insulin secretion (GSIS). Relative to basal conditions, glucose-stimulated ATP-levels were increased between 1.5 to 2-fold in both cell lines (Fig. 4C).

### Plasma membrane potential changes and cytoplasmic free Ca^2+^

Despite showing a similar fold increase in ATP levels in the presence of glucose, GSIS, expressed as fold-response, was lower in EndoC-βH1 cells. Since the plasma membrane potential (Δψ_p_) is largely controlled by the activity of K^+^_ATP_-channels, we examined whether the coupling of ATP to Δψ_p_ differed between the cell lines. To monitor changes in Δψ_p_, we used the fluorescent Δψ_p_ indicator, plasma membrane potential indicator, termed “PMPI”, the cellular uptake of which increases in response to plasma membrane depolarization [30].

**Fig 5:**
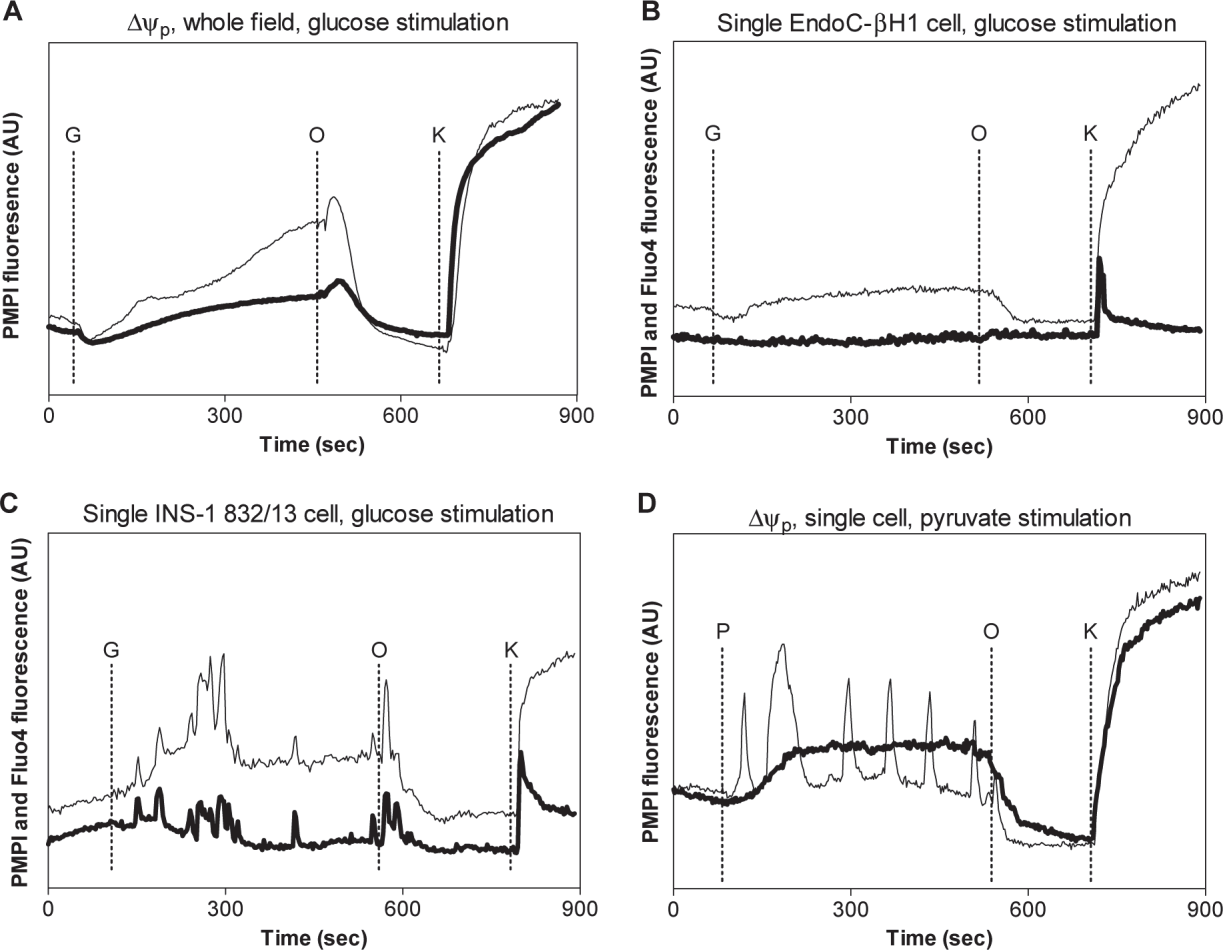
Plasma membrane potential and cytoplasmic free Ca^2+^ changes in EndoC-βH1 and INS-1 832/13 cells. Whole-field plasma membrane potential changes (A) in EndoC-βH1 (bold line) and INS-1 832/13 (thin line) cells. Additions: G, glucose, 16.7 mM; O, oligomycin, 0.5 ng/μL; K, KCl, 25 mM. Plasma membrane potential (thin line) and the free cytoplasmic Ca^2+^ (bold line) in (B) a single EndoC-βH1 cell and (C) a single INS-1 832/13 cell. (D) Representative single cell plasma membrane potential changes in response to pyruvate stimulation (P, 10 mM) in EndoC-βH1 (bold line) and INS-1 832/13 (thin line) cells. Data shown are representative for n = 3 experiments.

In basal conditions, EndoC-βH1 and INS-1 832/13 cells maintained a stable Δψ_p_. Since Δψ_p_ oscillations in dispersed clonal cells are not synchronized, we compared the field average depolarization of approximately 100 cells. In both cell lines, glucose initiated a slowly increasing depolarization (Fig. 5A). Inhibition of the mitochondrial ATP synthase with oligomycin resulted in repolarization as oxidative phosphorylation was inhibited. The repolarization was preceded by a brief enhanced depolarization. Although EndoC-βH1 cells responded to glucose by depolarizing, we failed to detect oscillations in individual cells, which were apparent in sub-populations of INS-1 832/13 cells. Accordingly, parallel monitoring of cytoplasmic free [Ca^2+^] showed the absence of [Ca^2+^] spiking in individual EndoC-βH1 cells (Fig. 5B). INS-1 832/13 cells showed a heterogeneous response to glucose stimulation, with some cells initiating Δψ_p_ oscillations and others progressively depolarizing without oscillations. Individual INS-1 832/13 cells showed a sustained depolarization in response to glucose, with a sub-population showing prolonged Δψ_p_ bursting and [Ca^2+^] spiking (Fig. 5C). Pyruvate stimulation induced a quick and sustained depolarization in EndoC-βH1 cells, again without oscillations, whereas a proportion of INS-1 832/13 cells oscillated. In both cell lines, oligomycin induced a repolarization before addition of KCl to calibrate the responses (Fig. 5D).

## Discussion

The stable human beta cell line, EndoC-βH1, realizes a much needed tool for detailed studies of human beta cell biology, circumventing the deficiency of sufficient amounts of primary human tissue. However, as previous studies on beta cell function mainly have been performed in rodent models, detailed knowledge on the human beta cells is still incomplete. To increase such knowledge, we compared metabolism in human EndoC-βH1 cells [12] with that in rat INS-1 832/13 cells [14,15].

Both cell lines showed robust viability and proliferation over time, although the proliferation rate of INS-1 832/13 cells was higher than that of EndoC-βH1 cells. Overall, our results revealed similar glucose-induced changes in insulin secretion, glucose utilization, metabolite profiles and respiratory rate in both cell lines, although the magnitudes of responses were lower in EndoC-βH1 cells. Depolarization with KCl induced additional insulin secretion, indicating that the exocytotic machinery in both cell lines appears to function normally. Although the amount of insulin released from EndoC-βH1 cells in response to glucose was greater, perhaps due to higher insulin content, the fold-response of GSIS in EndoC-βH1 cells was lower. This may be due to higher basal secretion of insulin, which is sometimes observed under pathological conditions. The lower rate of glucose utilization in EndoC-βH1 cells may reflect expression of GLUT1 instead of GLUT2, which is expressed in rodent beta cells, while the former predominates in human beta cells [9]. However, despite 10-fold higher glucose uptake via GLUT2, this is not expected to impact glycolytic rate as the rate of glucose uptake by GLUT1 and GLUT2 exceeds the rate of glucose phosphorylation by glucokinase (GCK) [9]. Moreover, EndoC-βH1 cells expressed only GCK while INS-1 832/13 cells expressed both hexokinase 1 (HK1) and GCK (S1 Table). Clearly, this had no impact on glycolytic rate, which was higher in INS-1 832/13 cells. In fact, contrary to what would be expected from a higher K_m_ glucose transport afforded by GCK, basal insulin secretion was higher in EndoC-βH1 cells.

In contrast to primary cell cultures, but in line with previous studies, both cell lines responded to pyruvate with increased insulin secretion and respiration [31–33]. If a beta cell is responsive to pyruvate, it implies that insulin would be released during exercise, as pyruvate and/or lactate are released from skeletal muscle. This would be physiologically detrimental. The molecular correlate of this normal “unresponsiveness” to pyruvate is the low expression level of monocarboxylate transporter (MCT/SLC16A1) in pancreatic beta cells [34]. A genetically determined exercise-induced hypoglycemia has been attributed to aberrant expression of MCT in beta cells [35]. Indeed, MCT is viewed as one of the archetypal “forbidden/disallowed” genes in the beta cell [36,37]. However, in clonal beta cells, MCT1 is constitutively expressed [31–33]. MCT1 was also expressed in EndoC-βH1 and INS-1 832/13 cells (S1 Table). The reason for expression of MCT1 in clonal beta cells is unclear. A possibility is that both are tumor cell lines, which need to survive in an environment with a limited supply of substrates as well as oxygen. It will thus be interesting to determine whether MCT1 expression decreases upon growth arrest of EndoC-βH1 cells [38].

To further investigate glucose-stimulated metabolic responses, metabolite profiling was performed at basal and stimulatory glucose levels. Overall, alterations in metabolite levels provoked by glucose were similar between the cell lines. We refrained from making comparisons between the different models since relative changes were determined. Minor changes in basal levels may therefore have a profound effect on the fold-response, yielding apparent differences, which may not relate to actual metabolite content. For regulatory purposes, however, changes in metabolite levels may still be highly relevant. Bearing this in mind, fold-changes in TCA-cycle intermediate levels seemed most vigorous in INS-1 832/13 cells followed by EndoC-βH1 cells and human islets. Observed differences could be species-specific rather than cell line specific. Metabolic rate has been suggested to decrease with increasing body size [39,40]. Generally, the glucose-induced increases in metabolites in human islets appeared lower than in the cell lines. This may be due to time preceding isolation and time in culture as well as interactions between the cell-types which form human islets. Intracellular lactate levels were found to be glucose-responsive in INS-1 832/13 and islets, confirming previous observations in cells [21], but glucose-unresponsive in EndoC-βH1 cells. Again, the islet source of lactate is unclear; non-beta cells may contribute to this release. In a previous study, lactate release, but not intracellular level, was found to parallel glucose unresponsiveness [17]. Here, we found that lactate release upon glucose stimulation was more pronounced in EndoC-βH1 cells.

Differences in metabolic responses may be due to alterations in mitochondrial metabolism or coupling of cytosolic and mitochondrial metabolism. A difference in mitochondrial metabolic flexibility is suggested since the relative stimulation in pyruvate-induced respiration was lower in EndoC-βH1. Hence, both glycolysis and respiration were less fuel-responsive in EndoC-βH1 compared to INS-1 832/13 cells.

Glucose-fueled respiration was associated with a lower relative proton leak in EndoC-βH1 cells and human islets compared to the INS-1 832/13 cells; as well as with pyruvate-fueled respiration in the cell lines. However, the overall coupling efficiency was the same in EndoC-βH1, INS-1 832/13 cells and human islets. In EndoC-βH1 cells, maximal respiration, obtained after FCCP addition, was blunted in response to glucose but not pyruvate. This blunted maximal respiration rate was also observed in the human islets. Maximal respiratory capacity in the presence of protonophore is dependent on processes ‘upstream’ of the mitochondrial proton circuit (transport, metabolism, electron transport etc.). The failure of the EndoC-βH1 cells to maintain respiration with elevated glucose following oligomycin and FCCP reflects a failure of glycolysis. This may be explained by cytosolic ATP depletion following addition of oligomycin, since pyruvate stimulated respiration, independent of cytosolic ATP, was enhanced under the same conditions.

Another possibility is decreased malate-aspartate- and/or glycerolphosphate-shuttle activity leading to decreased regeneration of cytoplasmic NAD^+^. This may cause EndoC-βH1 cells to "leak" glucose-metabolites towards lactate production, substituting for the role played by the two shuttles in NAD^+^ replenishment [41,42]. In accordance, metabolite profiling showed that two components of the malate-aspartate shuttle, aspartate and malate, were regulated by glucose in INS-1 832/13 cells, while only malate was increased in EndoC-βH1 cells. Clearly, differences in replenishment of cytosolic NAD^+^ via LDH and the malate-aspartate- and glycerolphosphate-shuttles, could impact glycolytic rate, mitochondrial metabolism and respiration and subsequently GSIS. The increased redirection of metabolites towards lactate production could also account, at least partially, for the discrepancy between the 6-fold increase in glycolytic rate and the 1.2-fold increase in respiration upon glucose stimulation in EndoC-βH1 cells; less reducing equivalents are produced for respiration when lactate is produced. It should be noted that lactate generation and release will decrease the bioenergetic responsiveness of cells to altered glucose availability, by allowing a Pasteur effect.

Plasma membrane depolarization and Ca^2+^ influx are crucial for normal insulin secretion. EndoC-βH1 as well as INS-1 832/13 cells exhibited depolarization of the plasma membrane in response to glucose stimulation, an event also seen in human islets [43]. Surprisingly, the slow plasma membrane depolarization of individual EndoC-βH1 cells triggered neither oscillations nor Ca^2+^ spiking. There is close parallelism between individual Δψ_p_ and Ca^2+^ oscillations, since generally each depolarization induces a concomitant rise in the cytosolic Ca^2+^ concentration, which is the triggering signal for insulin secretion [44], seen also in human islets [45,46]. Expression of two VDAC differed between EndoC-βH1 and INS-1 832/13 cells but no significant differences were found between EndoC-βH1 cells and human islets. The absence of oscillations in the EndoC-βH1 cells needs further investigation to better understand the underlying processes. This lack of Ca^2+^ spiking may support the notion of lower NADH shuttle activity and explain the lower TCA-cycle activity and insulin response observed in EndoC-βH1 cells. Activation of the malate-aspartate shuttle [47] as well as TCA-cycle dehydrogenases have been shown to depend on Ca^2+^ [5].

Although differences were observed in stimulus-secretion coupling between the two cell lines, the overall metabolic function was similar. Hence, previous knowledge on beta cell metabolism largely translates from studies in the INS-1 832/13 cell line to the human EndoC-βH1 cell line. It is difficult to resolve whether the overall lower metabolic and proliferative rates in EndoC-βH1 cells were due to the fact that humans have a lower metabolic rate than rodents, or whether different protocols used for cell immortalization as well as culture conditions played a role. To resolve this issue, sorted primary beta cells from humans and rodents would be helpful. However, many of the experiments performed here are not feasible in sorted primary cells. This notwithstanding, our analyses showed that EndoC-βH1 cells are as useful as the INS-1 cell lines, with the advantage that human genetics of T2D now can be directly applied to an *in vitro* model. In addition, this model is amenable to genetic and functional manipulations, such as the study of the impact of single nucleotide polymorphisms, methylations, and histone modifications. In summary, the EndoC-βH1 cell line may become a bridge between the abundant rodent *in vitro* models and the scarce primary human material. This may resolve some of the known and yet unknown species-dependent differences between rodents and humans that hamper understanding of T2D pathogenesis.

## Supporting Information

S1 Fig Doubling times and viability in EndoC-βH1 and INS-1 832/13 cell lines. Doubling times (solid symbols, left y-axis) based viable cell numbers and cell viability (open symbols, right y-axis) for EndoC-βH1 (squares) and INS-1 832/13 (circles) cells as a function of passage number post thaw.
(TIF)

S2 Fig Glucose stimulated insulin secretion in EndoC-βH1 cells, INS-1 832/13 cells and human islets. Glucose stimulated insulin secretion in EndoC-βH1 cells (white bar), INS-1 832/13 cells (black bar) and human islets (grey bar) expressed as the mean of the fold to basal from each biological replicate as opposed to the fold of the averaged basal and averaged stimulated levels (Fig. 1). Data are expressed as mean ±S.E.M. Differences between conditions were evaluated as described in the methods section. *p<0.05.
(TIFF)

S3 Fig Expression levels of voltage dependent calcium channels in EndoC-βH1, INS-1 832/13 cells and human islets. qPCR measurements of mRNA expression levels of CACNA1A, CACNA1C, CACNA1D and CACNA1H in EndoC-βH1 (white bars), INS-1 832/13 (black bars) cells and human islets (grey bars). Data are expressed as mean ±S.E.M. Differences between conditions were evaluated as described in the methods section. *p<0.05, ***p<0.001.
(TIF)

S1 Table Transcriptomics data for EndoC-βH1 and INS-1 832/13 cells. Check marks indicate the gene was expressed in the cells and an X that it was not detected in the array.
(DOC)
