# Supplementary figures and images for: Characterization of Stimulus-Secretion Coupling in the Human Pancreatic EndoC-βH1 Beta Cell Line
Source: PLoS One. 2015 Mar 24;10(3):e0120879. doi: 10.1371/journal.pone.0120879 (PMC4372368; doi:10.1371/journal.pone.0120879)

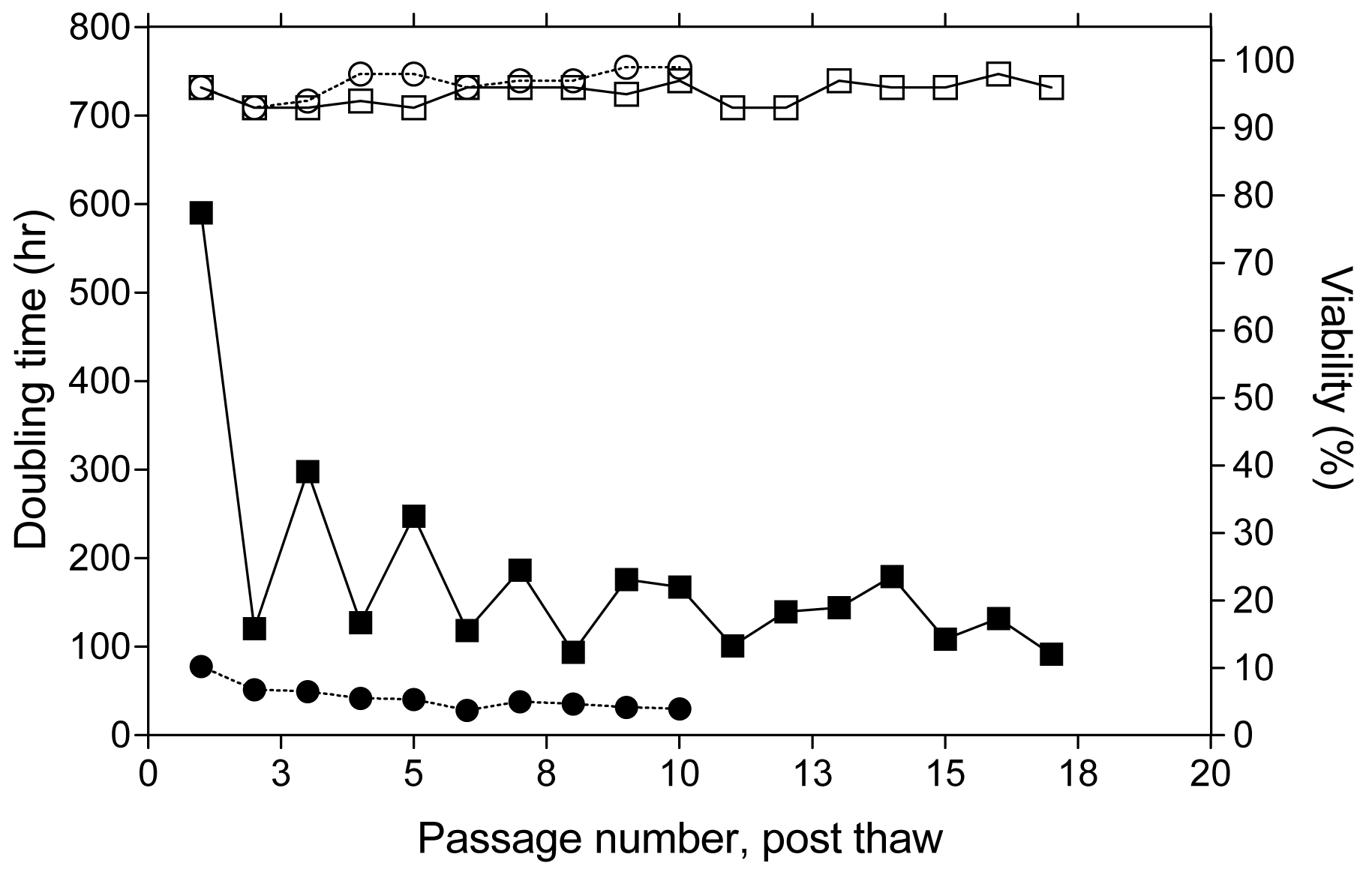

Supplement: S1 Fig — Doubling times (solid symbols, left y-axis) based viable cell numbers and cell viability (open symbols, right y-axis) for EndoC-βH1 (squares) and INS-1 832/13 (circles) cells as a function of passage number post thaw. (TIF) [file pone.0120879.s001.tif]

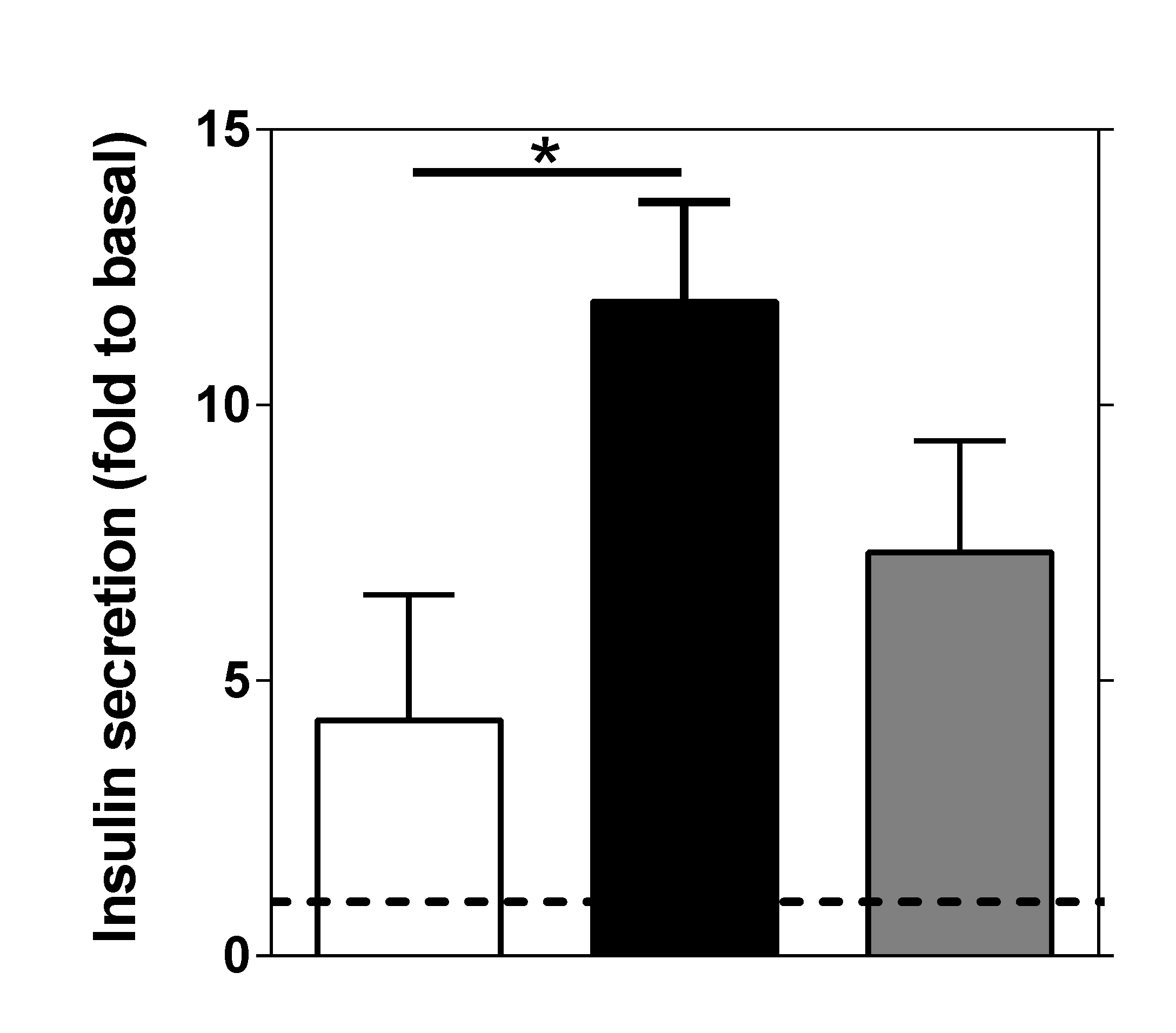

Supplement: S2 Fig — Glucose stimulated insulin secretion in EndoC-βH1 cells (white bar), INS-1 832/13 cells (black bar) and human islets (grey bar) expressed as the mean of the fold to basal from each biological replicate as opposed to the fold of the averaged basal and averaged stimulated levels (Fig. 1). Data are expressed as mean ±S.E.M. Differences between conditions were evaluated as described in the methods section. *p<0.05. (TIFF) [file pone.0120879.s002.tiff]
