# Supplementary material for: Characterization of Stimulus-Secretion Coupling in the Human Pancreatic EndoC-βH1 Beta Cell Line
Source: PLoS One. 2015 Mar 24;10(3):e0120879. doi: 10.1371/journal.pone.0120879 (PMC4372368; doi:10.1371/journal.pone.0120879)
Supplement: S1 Table — Check marks indicate the gene was expressed in the cells and an X that it was not detected in the array. (DOC) [file pone.0120879.s004.doc]

**S1 Table.** **Transcriptomics data for EndoC-βH1 and INS-1 832/13 cells.** Check marks indicate the gene was expressed in the cells and an X that it was not detected in the array.

| **Gene Name** | **Gene** | **EndoC-βH1** | **INS-1 832/13** |
| --- | --- | --- | --- |
| Glucose Transporter 1 | SLC2A1 | ✔ | ✔ |
| Glucose Transporter 2 | SLC2A2 | ✔ | ✔ |
| Glucose Transporter 4 | SLC2A4 | x | ✔ |
| Hexokinase I, II and III | HK 1, 2, 3 | x | ✔ |
| Glucokinase (Hexokinase IV) | GCK | ✔ | ✔ |
| Pyruvate carboxylase | PC | ✔ | ✔ |
| Pyruvate dehydrogenase, alpha 1 | PDHA1 | ✔ | ✔ |
| Pyruvate dehydrogenase, beta | PDHB | ✔ | ✔ |
| Monocarboxylate transporter 1 | SLC16A1 | ✔ | ✔ |
| Lactate dehydrogenase A | LDHA | ✔ | ✔ |
| Lactate dehydrogenase B | LDHB | ✔ | ✔ |
| Lactate dehydrogenase C | LDHC | x | ✔ |
| Lactate dehydrogenase D | LDHD | Unclear | ✔ |
| Transaldolase | TALDO1 | ✔ | ✔ |
| Phosphogluconate dehydrogenase | PGD | ✔ | ✔ |
| Solute carrier family 1, member 3 | SLC1A3 | x | ✔ |
| Malate dehydrogenase 1 | MDH1 | ✔ | ✔ |
| Malate dehydrogenase 2 | MDH2 | ✔ | ✔ |
| Aspartate aminotransferase (soluble) | GOT1 | ✔ | ✔ |
| Aspartate aminotransferase (mitochondrial) | GOT2 | ✔ | ✔ |
| Glycerol-3-phosphate dehydrogenase 1 (soluble) | GPD1 | ✔ | ✔ |
| Glycerol-3-phosphate dehydrogenase 2 (mitochondrial) | GPD2 | ✔ | ✔ |
| Phosphoenolpyruvate carboxykinase 1 (soluble) | PCK1 | x | ✔ |
| Phosphoenolpyruvate carboxykinase 2 (mitochondrial) | PCK2 | ✔ | ✔ |
| 3-oxoacid CoA transferase 1 | OXCT1 | ✔ | ✔ |
| Acetoacetyl-CoA synthetase | AACS | ✔ | ✔ |
| Solute carrier family 25, member 11 | SLC25A11 | ✔ | ✔ |
| Solute carrier family 25, member 12 | SLC25A12 | ✔ | ✔ |
| Transcription factor A (mitochondrial) | TFAM | ✔ | ✔ |
| Transcription factor B1 (mitochondrial) | TFB1M | ✔ | ✔ |
| Transcription factor B2 (mitochondrial) | TFB2M | ✔ | ✔ |
| Hexose-6-phosphate dehydrogenase | H6PD | ✔ | ✔ |
| Glucose-6-phosphatase, catalytic subunit | G6PC | x | ✔ |
| Glucose-6-phosphatase, catalytic subunit 2 | G6PC2 | ✔ | x |
| Glucose-6-phosphatase, catalytic subunit 3 | G6PC3 | Unclear | ✔ |
| Phosphofructokinase | PFK L/M/P | ✔ | ✔ |
| Fructose-1,6-bisphosphatase, Aldolase B | ALDOB | x | ✔ |
